# Incidence, risk factors, and outcomes of anti-tuberculosis drug-induced hepatotoxicity in people with and without HIV in Thailand and the Philippines

**DOI:** 10.2478/abm-2026-0020

**Published:** 2026-06-30

**Authors:** Ryan Christopher Geturbos Atuel, Emmanuel A. Gutierrez, Napon Hiranburana, Tanakorn Apornpong, Sasiwimol Ubolyam, Ma. Tarcela S. Gler, Anchalee Avihingsanon, Sivaporn Gatechompol, Stephen J. Kerr

**Affiliations:** School of Global Health, Faculty of Medicine, Chulalongkorn University, Bangkok 10330, Thailand; TB HIV Innovations and Clinical Research Corp., Cavite 4118, Philippines; HIV-NAT, Thai Red Cross AIDS and Infectious Diseases Research Centre, Bangkok 10330, Thailand; Division of Infectious Diseases, Faculty of Medicine, Ramathibodi Hospital, Mahidol University, Bangkok 10400, Thailand; Center of Excellence in Tuberculosis, Faculty of Medicine, Chulalongkorn University, Bangkok 10330, Thailand; Research Affairs, Faculty of Medicine, Chulalongkorn University, Bangkok 10330, Thailand; The Kirby Institute, University of New South Wales, Sydney 1002, Australia

**Keywords:** anti-tuberculosis therapy, drug-induced hepatotoxicity, HIV, TB-HIV, tuberculosis treatment

## Abstract

**Background:**

Anti-tuberculosis drug-induced hepatotoxicity (DIH) is a common complication among people living with HIV (PLWH), potentially leading to treatment interruptions.

**Objective:**

To determine the incidence of DIH and identify risk factors in Thai and Filipino PLWH on tuberculosis (TB) treatment, compared with those with TB mono-infection.

**Methods:**

We conducted a cohort study of Thai and Filipino adults with active TB using retrospective data from Thailand (2000–2024) and prospective data from the Philippines (2024). DIH was defined as AST or ALT ≥3 times the upper limit of normal. Logistic regression was used to assess DIH risk factors among PLWH; TB treatment outcomes were compared by DIH status.

**Results:**

Among 222 participants (138 PLWH, 84 HIV-negative), median age was 38 years, 63% were male, and median body mass index (BMI) was 18.2 kg/m^2^. DIH occurred in 13.8% of PLWH and 9.5% of HIV-negative individuals (*P* = 0.35), with median onset at 10 and 3 weeks, respectively. Among PLWH, hepatitis C co-infection (aOR 5.84, 95% CI 1.51–22.55, *P* = 0.01) and lower BMI (aOR 0.78/kg/m^2^, 95% CI 0.63–0.97, *P* = 0.03) were independently associated with DIH. Dolutegravir use showed a non-significantly lower DIH risk (OR 0.51, 95%CI, 0.18 – 1.42). TB treatment success was similar between those with and without DIH (92.6% vs 90.3%); mortality was slightly higher with DIH (3.7% vs 1.5%).

**Conclusions:**

Hepatitis C co-infection and low BMI are key risk factors for DIH among PLWH receiving TB treatment. Despite these risks, DIH did not significantly impact treatment outcomes.

Tuberculosis (TB) remains a significant global health threat, with an estimated 10.8 million new TB cases and 1.25 million deaths globally in 2023 [1]. Approximately 25% of the global population is estimated to have been infected with *Mycobacterium tuberculosis* [2], with 10% progressing to active TB disease during their lifetime [3]. People living with HIV (PLWH) face a 16-fold elevated risk of developing active TB, which remains the leading cause of death in this population [1]. Although antiretroviral therapy (ART) reduces TB incidence by approximately 65% across all CD4 count levels, PLWH remain at elevated risk of developing active TB even after ART initiation [4].

In 2023, most TB cases were located in Southeast Asia (45%), Africa (24%), and the Western Pacific (17%). Thailand and the Philippines are among the top countries with a high burden of TB and HIV co-infection. In addition to an increased mortality risk, the co-occurrence of TB and HIV poses unique treatment challenges. One key concern during TB treatment is drug-induced hepatotoxicity (DIH), a condition characterized by liver damage from antituberculosis medications [5]. Hepatotoxicity has a reported incidence ranging from 2% to 18%, and can lead to treatment interruptions, delays, or even discontinuation of TB therapy [6, 7]. The World Health Organization (WHO) and Thai national TB guidelines recommend standardized first-line TB treatment regimens for drug sensitive TB comprising isoniazid (H), rifampicin (R), pyrazinamide (Z), and ethambutol (E) [7, 8]. Among these, isoniazid, rifampicin, and pyrazinamide are known to be potentially hepatotoxic, and can lead to DIH [9].

While DIH has been well-documented, the existing literature primarily focuses on either human immunodeficiency virus (HIV)-negative or positive populations separately and rarely addresses risk factors for DIH among Asian PLWH. Known factors associated with DIH include the presence of extra-pulmonary TB, low body mass index (BMI), and elevated baseline liver enzyme levels [10]. Increasing evidence suggests that genetic predisposition is a critical determinant of hepatotoxicity, with N-acetyltransferase 2 (NAT2) acetylation status strongly influencing susceptibility. Slow acetylators have a threefold higher risk of anti-TB DIH and earlier onset compared with rapid or intermediate acetylators [11]. Among PLWH, additional risk factors for DIH include BMI <18.5 kg/m^2^, co-infection with hepatitis B and C, a low CD4 + cell count <50 cells/mm^3^, extra-pulmonary TB, and WHO HIV/AIDS stage 4 disease [10, 12, 13]. In this study, we aimed to assess the incidence of DIH in Asian PLWH with TB co-infection, compare this incidence to people with HIV-negative TB infection, and assess factors that were associated with developing DIH.

## Methods

### Study population

We conducted a cohort study among Thai and Filipino adults diagnosed with TB, using retrospective data from Thailand (March 2000–June 2024) and prospective data from the Philippines (June–November 2024). The retrospective data came from the HIV-NAT 006 long-term cohort (Clinicaltrials.gov NCT00411983), and an age- and sex-frequency matched cohort of HIV-negative controls from the Center of Excellence in TB, King Chulalongkorn Memorial Hospital. The prospective cohort enrolled newly diagnosed TB patients from two sites in Cavite, Philippines: a hospital-based TB referral clinic (Dasmarinas City Health Office) and a tertiary hospital with both TB and HIV clinics (De La Salle Medical and Health Sciences Institute TB-DOTS). Participants were categorized into two groups: those with HIV co-infection (TB-HIV group) and those with TB alone (TB group).

Participants were adults (≥18 years) who initiated first-line anti-TB therapy, and had liver function tests available before, and 2–10 weeks after initiating anti-TB treatment. PLWH were receiving ART with either efavirenz (EFV) or dolutegravir (DTG) based regimens. Individuals diagnosed with multi-drug resistant TB, or abnormal/missing baseline and follow-up (2–10 weeks post TB treatment) liver enzyme test were excluded. All participants had at least 6 months follow-up after treatment initiation to assess TB outcomes. Data from both retrospective and prospective cohorts included demographic and clinical characteristics, liver enzyme profiles, TB and HIV treatment regimens, and TB treatment outcomes. Data were de-identified and collected using standardized forms. In the Thai retrospective cohort, liver function tests were conducted more frequently to coincide with scheduled HIV study visits.

### Definitions and outcomes

TB disease included both bacteriologically confirmed (by smear microscopy, culture, or GeneXpert MTB/RIF) and clinically diagnosed TB based on radiologic evidence of TB without bacteriological confirmation, combined with good clinical response to antituberculosis treatment. Hepatitis B co-infection was defined as positive hepatitis B surface antigen, while hepatitis C co-infection was defined as a positive hepatitis C virus (HCV) antibody test.

The primary outcome was the incidence of DIH, defined as alanine aminotransferase (ALT) or aspartate aminotransferase (AST) >3 × the upper limit of normal (ULN). This was based on the Thai TB treatment guidelines, which define DIH as ALT or AST >3 × ULN with symptoms, such as jaundice, abdominal pain, lethargy, or nausea, or >5 × ULN without symptoms [8]. We used the lower range due to inconsistent reporting regarding the presence of symptoms.

Secondary objectives included comparing DIH incidence between PLWH and HIV-negative TB patients, identifying factors associated with the development of DIH, and comparing TB treatment outcomes by DIH status. TB treatment outcomes were defined according to the WHO guidelines, modified as follows: (1) Cured: Individuals with bacteriologically confirmed TB at the beginning of treatment who became smear- or culture-negative at the last month of treatment and on at least one prior occasion; (2) Treatment Completed: Individuals who completed treatment without evidence of failure but lacked bacteriological confirmation at the last month of treatment; (3) Treatment Success: The sum of Cured and Treatment Completed; (4) Treatment Failure: Individuals with sputum smear- or culture-positive results at month 5 or later during treatment; (5) Died: Individuals who died for any reason during the course of TB treatment; and (6) Lost to Follow-up: Individuals who did not return to the clinic to complete 6 months of treatment [14].

### Data management and analysis

Initial sample size was based on a precision of 95% confidence intervals (CI) around an assumed DIH incidence of 12% in PLWH; we aimed to estimate the DIH incidence with a precision of ±6%, which would require at least 113 participants.

The participants’ continuous data was described as median (interquartile range [IQR]), and categorical data as frequencies (%), overall and by HIV-status. Differences between these study groups were assessed with χ^2^ tests or Fisher’s exact tests for categorical variables and the Mann–Whitney U test for continuous variables. Relative risks and risk differences, with 95% CI, were estimated to assess the association between HIV status and the incidence of DIH, with formal comparisons based on the χ^2^ test.

To identify potential factors associated with the onset of DIH in the whole cohort, we used multivariable logistic regression. The potential predictor covariates considered included demographic factors (age, sex, and BMI), TB-related characteristics (pulmonary versus extrapulmonary TB, previous TB history). A separate analysis was conducted specifically for TB-HIV participants, which included the same potential predictor covariates, as well as the antiretroviral regimen used, and CD4 count at the start of TB treatment. Variables with *P*-values <0.15 in univariable analysis were adjusted for in multivariable models. NAT2 genotype and phenotypes were available in a subset of Thai PLWH. We conducted a sensitivity analysis in these participants to assess the potential role of NAT2 acetylation phenotype on the development of DIH.

Statistical analyses were conducted using Stata version 18.5 (StataCorp, College Station, TX, USA). The study was approved by the Institutional Review Board of Faculty of Medicine, Chulalongkorn University (COA No. 1154/2024) and Independent Ethics Committee of De La Salle Medical and Health Sciences Institute (IEC Code: 2024-21-01-A). All procedures were performed in accordance with local ethical guidelines and legal requirements, in full compliance with the Declaration of Helsinki, and approved by the relevant institutional review boards.

## Results

### Study cohort characteristics

**Figure 1.**
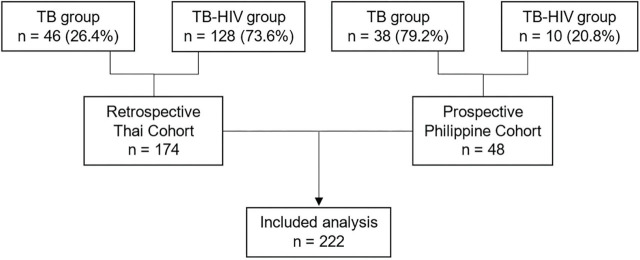
Overview of cases included in the analysis.

The study cohort included 222 participants: 174 from Thailand (128 in the TB-HIV group, 46 in the TB group) and 48 from the Philippines (10 in the TB-HIV group, 38 in the TB group), resulting in 138 and 84 participants in the TB-HIV and TB groups, respectively (**Figure 1**). Participant demographics are shown in **Table 1**. Males made up 63.1% of the cohort, with a higher proportion in the TB-HIV group (66.7%) than in the TB group (57.1%); median age was 38.2 (IQR 29–47) years. The median BMI was 18.2 (IQR 17.8–18.6) kg/m^2^ overall, with no significant difference between the TB group (18.2, IQR 17.8–18.6) and the TB-HIV group (19.9, IQR 19.4–20.5). Pulmonary TB was the most common TB type, and 8.6% of participants had a prior TB diagnosis, which was more frequent in the TB-HIV group (10.9%) than in the TB group (4.8%). A comparison of characteristics by country cohort is shown in **Supplementary Table 1**.

**Table 1.** Demographic characteristics

**Characteristic**	**TB (n = 84)**	**TB-HIV (n = 138)**	**Total (N = 222)**	***P***
Age (years), median (IQR)	40 (28–47)	37.3 (30.2–46.4)	38.2 (29–47)	0.997
BMI (kg/m^2^), median (IQR)	18.2 (17.8–18.6)	19.9 (19.4–20.5)	18.2 (17.8–18.6)	0.37
Sex				0.15
Female	36 (42.9)	46 (33.3)	82 (36.9)	
Male	48 (57.1)	92 (66.7)	140 (63.1)	
Type of TB**				
Pulmonary	71 (84.5)	105 (76.1)	176 (79.3)	0.17
Extrapulmonary	18 (21.4)	35 (25.4)	53 (23.9)	0.63
Prior TB diagnosis	4 (4.8)	15 (10.9)	19 (8.6)	0.14
Bacteriological confirmation**				
Sputum smear	2 (2.4)	18 (13.0)	20 (9.0)	-
Culture of M. tub.	31 (36.9)	20 (14.5)	51 (23.0)	-
Molecular testing (Gene Xpert)	48 (57.1)	81 (58.7)	129 (58.1)	-
Alcohol use	16 (19.0)	26 (18.8)	42 (18.9)	0.97
Illicit drug use	0 (0.0)	6 (4.3)	6 (2.7)	0.05
Comorbidities				
HBV co-infection	2 (2.4)	12 (8.7)	14 (6.3)	0.06
HCV co-infection	0 (0.0)	13 (9.4)	13 (5.9)	0.004
Diabetes mellitus	18 (21.7)	7 (5.1)	25 (11.3)	<0.001
Chronic kidney disease	2 (2.4)	1 (0.7)	3 (1.4)	0.30
Concomitant medications				
Fluconazole use	0 (0.0)	27 (19.6)	27 (12.2)	<0.001
Cotrimoxazole use	1 (1.2)	33 (23.9)	34 (15.3)	<0.001
Both (Cotrimoxazole + Fluconazole)	0 (0.0)	20 (14.5)	20 (9.0)	<0.001
NAT2 Genotype***				
Slow acetylator	-	38 (43.7)	38 (43.7)	-
Intermediate acetylator	-	25 (28.7)	25 (28.7)	-
Rapid acetylator	-	24 (27.6)	24 (27.6)	-
ART regimen				
Efavirenz	-	79 (57.2)	79 (57.2)	-
Dolutegravir	-	59 (42.8)	59 (42.8)	-
CD4 category (cells/mm^3^)				
>500	-	15 (10.9)	15 (10.9)	-
200–500	-	47 (34.1)	47 (34.1)	-
<200	-	76 (55.1)	76 (34.2)	-

* Data are n (%) unless otherwise specified.

** Data are not mutually exclusive.

The ***NAT2 genotype was available only for the Thai TB-HIV group.

BMI, body mass index; HBV, hepatitis B virus; HCV, hepatitis C virus; IQR, interquartile range; TB, tuberculosis.

Among those with bacteriological confirmation, molecular testing (GeneXpert) was the most frequently used diagnostic tool (58.1%), followed by culture (23.0%) and sputum smear (9.0%). Culture positivity was higher in the TB group (36.9%) than in the TB-HIV group (14.5%). Comorbidities varied between the groups: HCV co-infection was more common in the TB-HIV group (9.4%) than in the TB group (0%, *P* = 0.004), while diabetes mellitus was more prevalent in the TB group compared to the TB-HIV group (21.7% vs 5.1%) (*P* < 0.001). Alcohol use was similar between the groups (19.0% in the TB group vs 18.8% in the TB-HIV group) (*P* = 0.97), while illicit drug use was only reported in the TB-HIV group (4.3%, *P* = 0.053).

All ART use was exclusive to the TB-HIV group, with 57.2% taking efavirenz and 42.8% taking dolutegravir-based regimens. Concomitant medication use also differed significantly: fluconazole (19.6%) and cotrimoxazole (23.9%) were more commonly prescribed to the TB-HIV group (*P* < 0.001 for both). NAT2 genotyping was available in 87 participants in the TB-HIV group: 38 (43.7%) were slow acetylators, 25 (28.7%) were intermediate acetylators, and 24 (27.6%) were rapid acetylators.

### Incidence of drug induced hepatotoxicity between groups

The incidence of DIH was not significantly higher in the TB-HIV group compared to the TB group (13.8% vs 9.5%, *P* = 0.35, **Table 2**). The risk ratio for DIH in the TB-HIV group compared to the TB group was 1.45 (95% CI: 0.66–3.15), and the risk difference was 4.2% (95% CI: −4.3% to −12.8%). Notably, no Filipino participants developed DIH.

**Table 2.** Incidence of DIH in TB-HIV groups vs TB group

	**TB n (%)**	**TB-HIV n (%)**	**Total N (%)**
No DIH	76 (90.48)	119 (86.23)	195 (87.84)
DIH	8 (9.52)	19 (13.77)	27 (12.16)

DIH, drug-induced hepatotoxicity; TB, tuberculosis.

Among the 19 TB-HIV participants who developed DIH, 8 (42%) started ART before developing TB. The median time from ART initiation to anti-TB treatment in this group was 2.1 (IQR 0.8–14) years. Median onset to DIH after anti-TB treatment initiation in this group was 10 (IQR 6.5–20.5) weeks. The remaining 11 (58%) started anti-TB treatment prior to ART initiation, with a median interval of 2.7 weeks (IQR 2–10) between ART and anti-TB treatment initiation. Median onset to DIH after anti-TB treatment initiation in this group was 15 (IQR 4–28) weeks. Time to DIH onset differed by HIV-exposure group. In the TB-HIV group, the median onset was 10 weeks (IQR 5–28) after starting anti-TB therapy, compared to 3 weeks (2.5–5.5) in the TB group. Among TB-HIV participants, DIH onset was similar by ART regimen: 10 weeks (IQR 6–23) for those on efavirenz and 9.5 weeks (IQR 2–28) for those on dolutegravir.

### Factors associated with the development of DIH

In the overall cohort, multivariable logistic regression identified 3 factors that were independently associated with DIH: older age (adjusted odds ratio [aOR] 1.04, 95% CI 1.01–1.08; *P* = 0.02), lower BMI (aOR 0.77, 95% CI 0.65–0.92; *P* < 0.003), and HCV co-infection (aOR 6.13, 95% CI 1.60–23.45; *P* = 0.01).

In the TB-HIV subgroup, univariable logistic regression analysis identified lower BMI (OR 0.80, 95% CI 0.65–0.97; *P* = 0.02) and HCV co-infection (OR 4.60, 95% CI 1.32–16.03; *P* = 0.02) as significant risk factors for DIH in PLWH. Age, sex, prior TB diagnosis, and ART regimen and CD4 count were not significantly associated with DIH (**Table 3**). In a multivariable model, lower BMI (aOR 0.78, 95% CI 0.63–0.97; *P* = 0.03) and HCV co-infection (aOR 5.84, 95% CI 1.51–22.55; *P* = 0.01) remained independently associated with DIH.

**Table 3.** Risk factors for DIH during TB treatment

	**Univariable**		**Multivariable**	
	
	**Odds ratio (95% CI)**	***P***	**Adjusted odds ratio (95% CI)**	***P***
**TB-HIV and TB groups**				
TB-HIV co-infection vs TB mono-infection	1.62 (0.68–3.91)	0.28		
Age (per year increase)	1.03 (1.00–1.07)	0.05	1.04 (1.01–1.08)	0.02
BMI (kg/m^2^)	0.81 (0.70–0.95)	0.01	0.77 (0.65–0.92)	0.003
Male vs female	0.83 (0.37–1.89)	0.66		
Type of TB				
Pulmonary	1.58 (0.52–4.82)	0.42		
Extrapulmonary	0.52 (0.17–1.57)	0.25		
Prior TB diagnosis	0.84 (0.18–3.84)	0.82		
Alcohol use vs no alcohol use	0.97 (0.34–2.73)	0.95		
Illicit drug use vs no drug use	1.46 (0.16–13.00)	0.73		
HBV co-infection	1.21 (0.26–5.74)	0.81		
HCV co-infection	5.28 (1.59–17.57)	0.01	6.13 (1.60–23.45)	0.01
Diabetes mellitus	0.59 (0.13–2.68)	0.5		
Fluconazole use	1.3 (0.41–4.10)	0.65		
Cotrimoxazole use	1.3 (0.46–3.71)	0.62		
**TB-HIV group only**				
Age (per year increase)	1.01 (0.97–1.06)	0.52		
BMI (kg/m^2^)	0.8 (0.65–0.97)	0.02	0.78 (0.63–0.97)	0.03
Male vs female	1.22 (0.43–3.46)	0.71		
Type of TB		0.31		
Pulmonary	1.98 (0.54–7.26)	0.31		
Extrapulmonary	0.46 (0.12–1.69)	0.25		
Prior TB diagnosis	0.89 (0.18–4.29)	0.88		
ART regimen		0.20		
Efavirenz—NNRTI	1 (ref)			
Dolutegravir—INSTI	0.51 (0.18–1.42)			
CD4 category (cells/mm^3^)		0.39		
>500	1 (ref)			
200–500	3.41 (0.39–29.40)			
<200	2.10 (0.25–17.96)			
Alcohol use vs no alcohol use	1.29 (0.39–4.32)	0.68		
Illicit drug use vs no drug use	1.18 (0.13–10.68)	0.88		
HBV co-infection	1.19 (0.24–5.90)	0.83		
HCV co-infection	4.6 (1.32–16.03)	0.02	5.84 (1.51–22.55)	0.01
Diabetes mellitus	0.97 (0.11–8.56)	0.98		
Fluconazole use	1.08 (0.33–3.57)	0.90		
Cotrimoxazole use	0.97 (0.29–3.18)	0.96		

ART, antiretroviral therapy; BMI, body mass index; CI, confidence interval; DIH, drug induced hepatotoxicity; HBV, hepatitis B virus; HCV, hepatitis c virus; INSTI, integrase strand transfer inhibitor; NNRTI, non-nucleoside reverse transcriptase inhibitors; TB, tuberculosis.

### Sensitivity analysis for NAT2 acetylation phenotype and DIH risk

A sensitivity analysis was conducted among the Thai TB-HIV group to assess the influence of NAT2 acetylation phenotype on DIH risk. No significant association was identified between NAT2 phenotype and DIH occurrence, with 21/24 (12.5%) fast acetylators compared to 8/63 (12.7%) slow and intermediate acetylators developing DIH (*P* = 1.0).

### Tuberculosis treatment outcomes between groups

Among the 222 patients included in the analysis, the overall TB treatment success rate was 90.5% (**Table 4**). Treatment success was slightly lower in the TB-HIV group compared to the TB group (87.7% vs 95.2%). Mortality was also marginally higher among TB-HIV participants (2.2%) than in the TB group (1.2%), and a larger proportion of TB-HIV participants were not evaluated for treatment outcomes (7.2% vs 2.4%). However, these differences were not statistically significant (*P* = 0.44). Similarly, among those who developed DIH, treatment success remained high (92.6%) and comparable to those without DIH (90.3%). Mortality was slightly higher in the DIH group (3.7% vs 1.5%), though these differences were also not statistically significant (*P* = 0.83, **Table 4**). A total of 9 participants, 4 from the TB-HIV group and 5 from the TB group, underwent rechallenge with modified or alternative anti-TB regimens following DIH. All completed treatments without recurrence of hepatotoxicity achieved favorable outcomes.

**Table 4.** Tuberculosis treatment outcomes between groups

**Treatment outcome**	**TB**	**TB-HIV**	*P* *	**No DIH**	**DIH**	*P* *
	
	n (%)	n (%)		n (%)	n (%)	
Died	1 (1.2)	3 (2.2)	0.49	3 (1.5)	1 (3.7)	0.81
Lost to follow-up	0 (0.0)	1 (0.7)		1 (0.5)	0 (0.0)	
Not evaluated	2 (2.4)	10 (7.2)		11 (5.6)	1 (3.7)	
Treatment failed	1 (1.2)	3 (2.2)		4 (2.1)	0 (0.0)	
Treatment success	80 (95.2)	121 (87.7)		176 (90.3)	25 (92.6)	

* Fisher’s exact P.

DIH, drug-induced hepatotoxicity; TB, tuberculosis.

## Discussion

In this two-country cohort study of Thai and Filipino adults with drug-sensitive TB, we found that PLWH experienced a higher incidence of DIH compared to HIV-negative individuals. However, this difference was not statistically significant, and TB treatment outcomes were comparable between those who did and did not develop DIH. Importantly, low BMI and HCV co-infection consistently were independent predictors of DIH in both our TB-HIV and TB groups.

The overall incidence of DIH among PLWH in our cohort was 13.8%, aligning with previously reported ranges of 2%–28% among patients on first-line anti-TB therapy, particularly in HIV-endemic settings [15,16,17]. A systematic review and meta-analysis from India estimated a pooled DIH incidence of 12.6% [18]. We found a slightly higher incidence in the TB-HIV group than the TB-mono infection group, which reinforces concerns regarding the hepatic vulnerability of immunocompromised patients who may have a lower body weight. The observed incidence is consistent with other recent studies reporting hepatotoxicity rates ranging from 10% to 14% in TB/HIV co-infected populations [16, 17, 19]. Differences in DIH incidence across studies may be due to variations in hepatotoxicity definitions, diagnostic criteria, study populations, ethnic susceptibility influencing drug metabolism, and whether ART-related hepatotoxicity was accounted for in the analysis.

Interestingly, DIH occurred later among our TB-HIV cohort, with a median onset of 10 weeks, compared to 3 weeks in the TB-only group. Prior studies have typically reported hepatotoxicity within 4–6 weeks of initiating TB or ART. While early onset of DIH (within 6 weeks of anti-TB) has been highlighted in the literature [16, 17], the delayed onset of DIH in our cohort may be explained by 3 hypotheses. First, overlapping ART and anti-TB drug toxicities: of the 19 TB-HIV participants with DIH, 8 were already on long-term ART before initiating anti-TB therapy, and the remaining 11 initiated ART at a median of 2.7 weeks (IQR 2–10) after TB treatment initiation. Efavirenz has been linked to immune-mediated liver injury occurring within the first few months of treatment, and although dolutegravir is considered safer, liver injury with associated hypersensitivity has been reported [20, 21]. A prospective study in Ethiopian TB-HIV patients found a tenfold increased DIH risk with concurrent ART and anti-TB therapy [22]. Second, immune reconstitution inflammatory syndrome (IRIS) may have contributed to hepatotoxicity risk. Dolutegravir-based ART promotes robust viral suppression, and has been associated with increased IRIS risk in previous cohort studies [23, 24], reinforcing the need for early hepatotoxicity screening and individualized regimen adjustments [25]. Third, the detection of DIH may be confounded by the duration of liver function test monitoring after ART initiation. The majority of the HIV-NAT cohort TB-HIV participants were asymptomatic, and our DIH event occurred at a median of 10 weeks. In contrast, there were only 10 Filipino TB-HIV participants who prospectively enrolled, and no DIH was observed among them. In addition, no DIH was found in 38 Filipino TB-mono participants. This discrepancy may partly relate to the monitoring schedule in this cohort which involved one blood draw between 2 and 10 weeks. However, it is important to note that the upper 97.5% CI around a zero-incidence count in the 10 Filipino PLWH and 38 TB mono-infections would be 30.8% and 9.3%, respectively, which is consistent with the observed incidence in Thai participants in this study, and also consistent with what might be observed with incidence rates of 2%–28% reported in other settings [15,16,17].

Age was a significant risk factor for DIH in our entire cohort, consistent with guidelines noting increased susceptibility to hepatotoxicity among older adults due to an age-related decline in hepatic metabolic capacity, reduced drug clearance, and increased oxidative stress, particularly in patients over 50 years [26]. However, this association was not observed within our TB-HIV subgroup, possibly reflecting the age distribution differences between our TB-HIV and TB cohorts.

Low BMI was identified as an independent risk factor for DIH in both TB-only and TB-HIV study groups. This finding aligns with previous studies from Ethiopia and China showing a significantly higher incidence of DIH among TB/HIV co-infected patients with a BMI below 18.5 kg/m^2^ [27]. Malnutrition, as reflected by low BMI, likely impairs hepatic glutathione stores, thereby weakening antioxidant defenses and increasing susceptibility to oxidative stress, a key mechanism in hepatocellular injury. Low BMI may compromise hepatic reserve, limit drug metabolism capacity, and exacerbate toxicity from first-line anti-TB drugs [10, 16, 28].

HCV co-infection was also an independent risk factor for DIH in our cohort, although only seen in the TB-HIV group, because none of the participants in the TB group were known to have HCV infection. HCV co-infection exacerbates hepatic inflammation and impairs drug metabolism, thereby increasing susceptibility to hepatotoxicity during anti-TB treatment [29]. A systematic review and meta-analysis reported a pooled DIH incidence of 39.19% among patients with HCV co-infection [30], and one study found the risk of hepatotoxicity was 14.4 times higher in patients co-infected with HIV and HCV [31]. HCV-induced oxidative stress and impaired hepatic reserve heighten vulnerability to hepatocellular damage and fibrosis progression [32, 33]. Given the synergistic hepatotoxic effects of TB drugs and chronic viral hepatitis, routine viral hepatitis screening, frequent liver function monitoring, and potential antiviral therapy integration are essential in TB-HIV co-infected patients [34]. Even with a relatively low prevalence of HCV in our cohort, its strong association with DIH emphasizes the critical need for routine viral hepatitis screening prior to anti-TB therapy, particularly in PLWH, where hepatic comorbidities are prevalent and may compound treatment-related risks.

Other examined variables—including sex, TB classification, CD4 cell count, alcohol use, HBV co-infection, diabetes mellitus, fluconazole, and cotrimoxazole use- did not show significant associations with DIH in this cohort. While previous studies have implicated fluconazole & cotrimoxazole in hepatotoxicity among PLWH [35, 36], their use was not independently associated with DIH in our study population, possibly due to limited exposure or effective clinical monitoring. Similarly, although low CD4 counts (<200 cells/mm^3^) reflect advanced immunosuppression, we did not find a significant correlation with DIH risk, aligning with previous studies [37].

Furthermore, the absence of significant associations with sex, alcohol use, and HBV co-infection is consistent with findings from previous studies in TB/HIV co-infected patients [38,39,40]. The absence of significant associations in our study may be partially explained by the limited sample size, reducing statistical power, or by effective clinical management of opportunistic infections and concomitant medications, which may have mitigated hepatotoxic risk.

Our sensitivity analysis among Thai participants with TB-HIV, NAT2 acetylation phenotype, was not significantly associated with DIH, partly due to a relatively small sample size. While slow NAT2 acetylation are typically considered at increased susceptibility to isoniazid-induced hepatotoxicity [11, 41], our findings indicate that in this population, other factors, such as HIV-associated hepatic dysfunction or additional metabolic, immunological, and clinical factors, may play a more dominant role in DIH susceptibility within this cohort. This emphasizes the need for larger-scale pharmacogenetic studies in TB-HIV co-infected populations to clarify the role of NAT2 in hepatotoxic risk stratification.

Treatment outcomes were favorable, with an overall treatment success rate of 90.5%, with slightly lower success among the TB-HIV than the TB-only group (87.7% vs. 95.2%) and among those who developed DIH compared to those who did not (90.3% vs. 92.6%). This aligns with findings from other studies. A nested case-control study in Ethiopia reported an 11.5% incidence of DIH but did not find a significant impact on treatment completion or success when patients were closely monitored and managed during the intensive phase of TB therapy [19, 39]. Similarly, a South African retrospective study observed that successful TB treatment can still be achieved in TB-HIV patients with DIH, particularly when drug rechallenge protocols are applied after liver injury resolution [42, 43]. While DIH remains a critical concern in TB-HIV co-infection, our findings suggest that with timely intervention and appropriate therapeutic adjustments, treatment success can still be achieved, reinforcing the importance of proactive monitoring and tailored clinical approaches.

Our study has several limitations. First, the relatively small number of DIH events limited statistical power to detect modest associations with some clinical variables. Second, TB-mono patients are not routinely screened for HCV co-infection, which may have led to a misclassification of some TB mono-participants as being HCV negative when they were in fact HCV positive. However, while not impossible, this scenario is unlikely as the TB prevalence in the Thai general population is 0.39%, but 22-fold higher in PLWH [44].

Third, NAT2 genotyping was performed only in the TB-HIV group, limiting cross-group comparisons of acetylator phenotype. Fourth, systematic longitudinal monitoring of liver function tests and standardized assessments of medication adherence were not consistently available across both study sites. Fifth, the absence of pharmacokinetic data, such as serum drug levels or metabolite profiling, limited our ability to explore potential associations between drug exposure and hepatotoxicity. Consequently, the precision of determining the exact timing, severity, and potential pharmacologic mechanisms of DIH may have been affected. These limitations are inherent to the retrospective nature of the study and highlight the need for future prospective research incorporating more robust pharmacologic and adherence assessments. Despite these limitations, our study provides additional evidence on the incidence of DIH in PLWH in Southeast Asia.

## Conclusion

In this Asian cohort, DIH developed in 13.8% of TB-HIV participants and 9.5% of TB-only participants, but treatment success remained high in both groups. HCV co-infection and low BMI were independently associated with increased DIH risk. These findings underscore the importance of targeted liver function monitoring and individualized care for high-risk patients initiating anti-TB therapy, particularly those with HIV and hepatic comorbidities.

## Notes

**Supplementary Table 1.** Demographic characteristics by study site (Thai vs Filipino)

**Characteristic**	**Thai (*n* = 174)**	**Filipino (*n* = 48)**	**Total (*N* = 222)**	***P***
Age (years), median (IQR)	40 (37–43)	32.5 (28–37)	38.2 (29–47)	<0.001
BMI (kg/m^2^), median (IQR)	20.1 (19.6–20.7)	19.4 (18.2–21.5)	18.2 (17.8–18.6)	0.29
Sex				0.56
Female	66 (37.9)	16 (33.3)	82 (36.9)	
Male	108 (62.1)	32 (66.7)	140 (63.1)	-
Type of TB**				<0.001
Pulmonary	128 (73.6)	48 (100.0)	176 (79.3)	-
Extrapulmonary	53 (30.5)	0 (0.0)	53 (23.9)	-
Prior TB diagnosis	16 (9.2)	3 (6.2)	19 (8.6)	0.52
Bacteriological confirmation**				0.02
Sputum Smear	20 (11.5)	0 (0.0)	20 (9.0)	-
Culture of M. tub.	44 (25.3)	7 (14.6)	51 (23.0)	-
Molecular Testing (Gene Xpert)	94 (54.0)	35 (72.9)	129 (58.1)	-
Alcohol use	26 (14.9)	16 (33.3)	42 (18.9)	0.004
Illicit drug use	6 (3.4)	0 (0.0)	6 (2.7)	0.19
Comorbidities				
HBV co-infection	14 (8.0)	0 (0.0)	14 (6.3)	0.05
HCV co-infection	13 (7.5)	0 (0.0)	13 (5.9)	0.05
Diabetes mellitus	14 (8.0)	11 (23.4)	25 (11.3)	0.003
Chronic kidney disease	3 (1.7)	0 (0.0)	3 (1.4)	0.36
Concomitant medications				
Fluconazole use	26 (14.9)	1 (2.1)	27 (12.2)	0.02
Cotrimoxazole use	28 (16.1)	6 (12.5)	34 (15.3)	0.51
Cotrimoxazole + Fluconazole	20 (11.5)	0 (0.0)	20 (9.0)	0.02

**HIV-specific variables**	**Thai (n = 128)**	**Filipino (n = 10)**	**Total (N = 138)**	
ART regimen				0.13
Efavirenz	71 (55.5)	8 (80.0)	79 (57.2)	-
Dolutegravir	57 (44.5)	2 (20.0)	59 (42.8)	-
CD4 category (cells/mm^3^)				0.07
>500	15 (11.7)	0 (0.0)	15 (10.9)	-
200–500	46 (35.9)	1 (10.0)	47 (34.1)	-
<200	67 (52.3)	9 (90.0)	76 (34.2)	-
NAT2 genotype***				
Slow acetylator	38 (43.7)	-	38 (43.7)	-
Intermediate acetylator	25 (28.7)	-	25 (28.7)	-
Rapid acetylator	24 (27.6)	-	24 (27.6)	-

* Data are n (%) unless otherwise specified.

** Data are not mutually exclusive.

*** NAT2 genotypes were available only for 87 Thai PLWH.

ART, antiretroviral therapy; BMI, body mass index; HBV, hepatitis B virus; HCV, hepatitis c virus; IQR, interquartile range; PLWH, people living with HIV; TB, tuberculosis.
